# Comparison of the therapeutic outcomes between open plantar fascia release and percutaneous radiofrequency ablation in the treatment of intractable plantar fasciitis

**DOI:** 10.1186/s13018-020-1582-2

**Published:** 2020-02-18

**Authors:** Yusong Yuan, Yuan Qian, Hao Lu, Yuhui Kou, Yangbo Xu, Hailin Xu

**Affiliations:** 1 Department of Trauma and Orthopedics, Peking University People’s Hospital, Peking University, 11th Xizhimen South Street, Beijing, China; 2 Department of Trauma Surgery, The Affiliated Hospital of Qingdao University, Qingdao, Shandong China; 3 Diabetic Foot Treatment Center, Peking University People’s Hospital, Peking University, Beijing, China; 4 Department of Bone and Joint Surgery, Affiliated Hospital of Southwest Medical University, Chongqing, China

**Keywords:** Intractable plantar fasciitis, Open plantar fascia release, Percutaneous radiofrequency ablation

## Abstract

**Background:**

Heel pain is one of the most common complaints in foot and ankle clinic, and one of the leading causes of heel pain is plantar fasciitis.

**Methods:**

A retrospective analysis was carried out in 31 cases (39 feet) of patients with intractable plantar fasciitis. In the enrolled 26 cases, 16 patients (19 feet) received open plantar fascia release, and the other 15 patients (20 feet) received percutaneous radiofrequency ablation. The surgical results were assessed by visual analog scale (VAS) and American Orthopaedic Foot and Ankle Society Ankle-Hindfoot Scale (AOFAS-AH) before and after surgery in all patients.

**Results:**

All 31 patients were followed up successfully, with a mean follow-up time of 58.77 months. There were no differences of patient’s demographics and characteristics information between the two groups. The average operative time of the feet in the open plantar fascia release is longer than that in the percutaneous radiofrequency ablation. Furthermore, the percutaneous radiofrequency ablation group had a shorter recovery time to normal activity than the open plantar fascia release group. There were no differences of postoperative VAS scores and the AOFAS-AH scores between the two groups. All patients reported satisfaction after either operation.

**Conclusion:**

The symptoms of pain and limb function were significantly improved in patients both of the partial plantar fascia release treated group and the percutaneous radiofrequency ablation treated group. The two types of surgical procedures shared the same long-term curative effects. However, percutaneous radiofrequency ablation was a better technique from the point of shorter operative time and postoperative recovery time.

**Trial registration:**

Retrospectively registered.

Heel pain is one of the most common problems in which patients are seeking help in the foot and ankle surgery clinic. Plantar fasciitis is one of the main pathologies in heel pain in adults and accounts for 11 to 15% of all foot diseases that require medical attention [1, 2]. Plantar fasciitis is more prevalent in sedentary individuals but also in athletes involving running sports [3].

The pathogenesis of plantar fasciitis is not clear. Lemont et al. believed that plantar fasciitis was a pathological change dominated by metatarsal fascia degeneration [4]. Nery et al. thought that heel pain was due to chronic strain of the metatarsal fascia, which was secondary to aseptic inflammation caused by microtear caused by repeated microinjury [5]. Most of the patients were satisfied with the conservative treatment, such as traction stretching exercise, splinting, bracing, extracorporeal shock wave, and oral administration of anti-inflammatory drugs no matter what the pathological changes were [6, 7]. Surgical interventions would be considered when patients with intractable plantar fasciitis showed negative responses to over 6 months of conservative treatment [8, 9]. There are many options for surgical treatment. Open surgery, endoscopic plantar fascia debridement, laser, platelet-rich plasma injection, radiofrequency ablation, and other programs have achieved a certain degree of curative effect [10–13].

The plantar fascia release and percutaneous radiofrequency ablation used in this study belong to minimally invasive surgery, but the difference between the two is not clear. Here is a retrospective analysis which was carried out to compare the therapeutic effects of open plantar fascia release and percutaneous radiofrequency ablation in the treatment of intractable plantar aponeurositis in order to pursue a better treatment of metatarsal fasciitis.

## Materials and methods

### Patients

A retrospective analysis was carried out in intractable plantar fasciitis patients who received surgical treatment in the Department of Orthopedic Surgery of our hospital during March 2009 to July 2018. Diagnosis of plantar fasciitis was made according to the guidelines described by McPoil et al. for plantar fasciitis including tenderness in the plantar medial heel region on palpation, pain most noticeable with initial steps after a period of inactivity but also worse following prolonged weight-bearing, and pain often precipitated by a recent increase in weight-bearing activity [14, 15]. The patients who suffered intractable plantar aponeurositis still felt stubborn heel pain after 6 months of conservative treatment.

### Inclusion criteria and exclusion criteria

All the patients were chosen by the following criteria. The inclusion criteria were patients diagnosed with plantar fasciitis, patients with failure of conservative treatment for at least 6 months, and patients without flatfeet or gastrocnemius contracture. The exclusion criteria were patients with any previous history of surgery for heel pain, associated pathology involving the lower limb such as history of tarsal tunnel syndrome, effusion of the ankle indicating an intra-articular disease, Achilles tendinopathy, patients with systemic disorder like diabetes mellitus and rheumatoid arthritis, and any recent history of aspirin or aspirin-like drug intake.

With the inclusion criteria and exclusion criteria, 31 cases operated by a single doctor were selected. The patient chose the surgical procedure according to their own wishes after communicating with the doctor. Among the enrolled 31 cases, 16 patients (19 feet) received open plantar fascia release, and the other 15 patients (20 feet) received percutaneous radiofrequency ablation. All the included cases were confirmed to have plantar fasciitis clinically. All patients received and failed at least 6 months of conservative treatment including stretching traction training, splinting, bracing, and extracorporeal shock wave.

### Surgical procedure

#### Open plantar fascia release

**Fig. 1:**
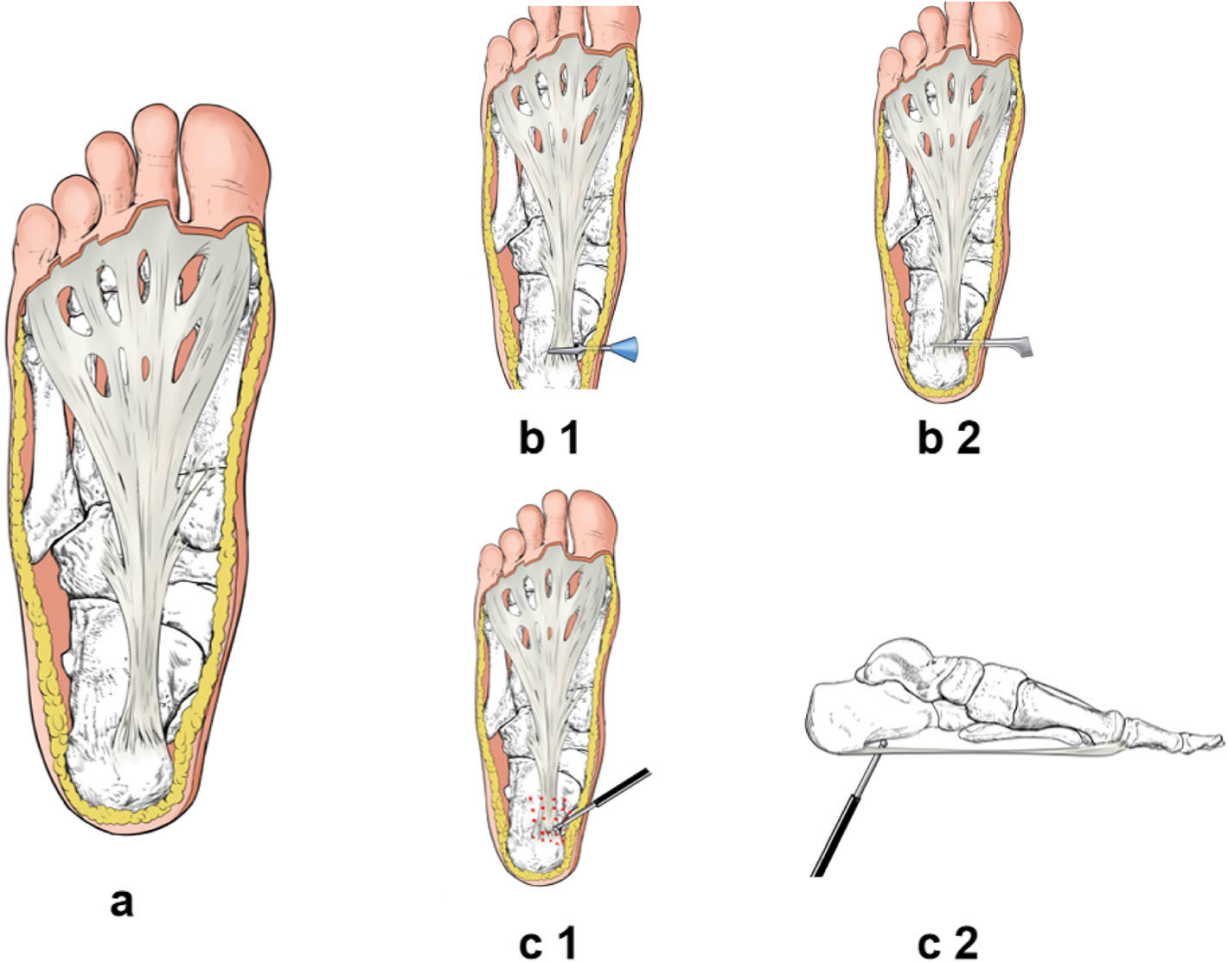
**a** An anatomical view of the tendon fascia. **b1**, **b2** A schematic view of percutaneous fascia surgery. **b1** The separation of the fascia with a stent. **b2** The iridium fascia is cut by 1/2 along the stent groove with a blade. **c1**, **c2** The surgical intent of radiofrequency ablation. The red dot in **c1** is the position at which the ablation needle is inserted. **c2** The depth at which the ablation needle is inserted

**Fig. 2:**
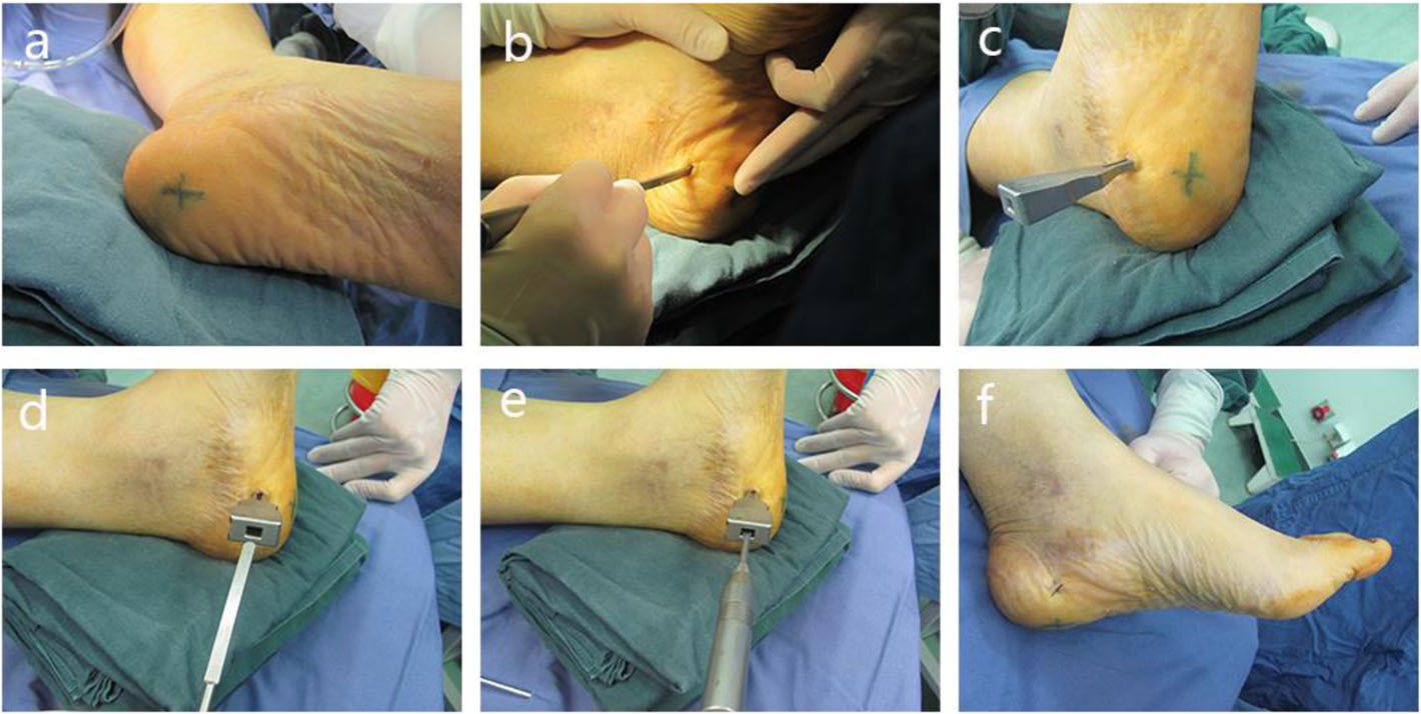
The procedure of open plantar fascia release. **a** We marked the pain zone at first. **b**–**f** Dissection carried out through the skin and subcutaneous tissue and exposed the plantar fascia from medial hindfoot. Half of the medial plantar fascia was released through medial incision. **e** There was only a micro-wound left on the foot

Patients who received open plantar fascia release were administered with sedation and regional block and placed in supine position. Patients were prepped and draped with standard routine iodine and alcohol and sterilized drapes. Tourniquet was used in 16 cases of patients (19 feet). One centimeter medial incision of the heel was used. Dissection was carried out through the skin and subcutaneous tissue and exposed the plantar fascia from medial hindfoot. Half of the medial plantar fascia was released (Figs. 1 and 2). Surgical wound was closed with suture following irrigation and covered with sterile dressings.

#### Percutaneous radiofrequency ablation

**Fig. 3:**
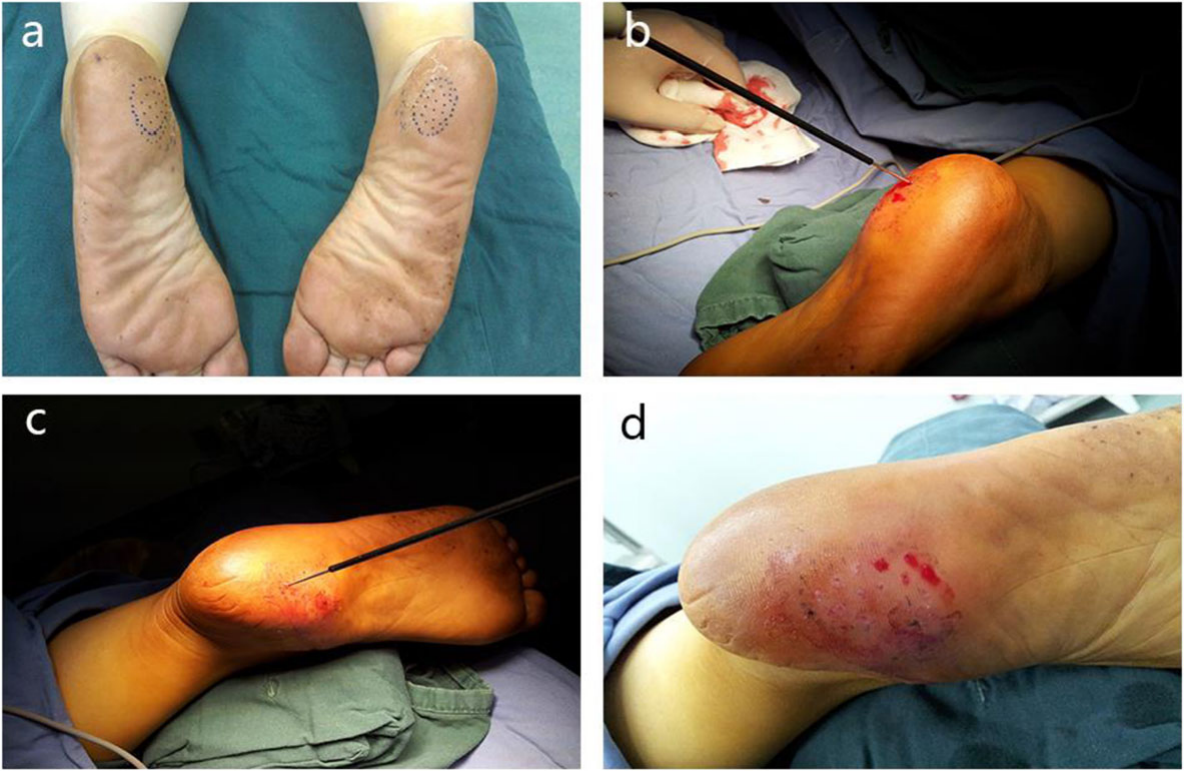
The procedure of percutaneous radiofrequency ablation. **a** We marked the pain zone the at first. **b**, **c** A 2-mm Kirschner wire was used as a puncture needle to puncture the skin and subcutaneous tissue at the marking points. Subsequently, a radiofrequency cutter was inserted to the plantar fascia level at each puncture point of the marked grid, and radiofrequency ablation was then performed percutaneously. **d** There was no obvious wound after operation

The maximum tenderness area of the affected side of the plantar fascia was identified preoperatively. Accordingly, we marked the tenderness area with a medical marker in a grid pattern with 3-mm intervals of points covering the entire mapped out area. Standard routine iodine and alcohol disinfection of skin prep and sterilized draping were applied. Local infiltration anesthesia was conducted using 1% lidocaine at the peripheral of the marked region followed by subcutaneous injection of normal saline at the marked region. A 2-mm Kirschner wire was used as a puncture needle to puncture the skin and subcutaneous tissue at the marking points. The puncture needle was stopped when the surgeon felt penetrating sense. Subsequently, a radiofrequency cutter was inserted to the plantar fascia level at each puncture point of the marked grid, and radiofrequency ablation was then performed percutaneously. Sterilized dressing was then applied locally at the end of the procedure (Figs. 1 and 3).

### Post-procedure protocol

Local soft sterile dressings were applied for all the patients and left on for 2 weeks. The patients were allowed to do mild active stretching exercises for the plantar fascia and Achilles tendon with the protection of a brace. High-impact activities were forbidden for 1 month.

### Assessment

Visual analog scale (VAS) and American Orthopaedic Foot and Ankle Society Ankle-Hindfoot Scale (AOFAS-AH) were adopted for assessment before and at 3 days after surgery in all patients to evaluate the pain and lower limb function of the studied patients before and after the operation. Sex, age, body mass index, and duration of pain were also recorded. All patients were followed up for patient-reported satisfaction rate with the curative effect of the procedures in outpatient visits at 1 month, 2 months, 3 months, and 12 months postoperation and in July 2018 at the end of this study.

### Statistical analysis

Both feet of the patients were analyzed using the bilateral data analysis. The SPSS 19.0 statistical software was used for statistical analysis. Continuous variables were presented as mean ± standard deviation (SD) and statistically analyzed by Student’s *t* test with normal distribution. Non-normally distributed data was analyzed by the Mann-Whitney *U* rank sum test. A value of *P* < 0.05 was considered as significant.

## Results

From March 2009 to July 2018, a total of 31 cases (39 feet) of intractable plantar fasciitis received surgical treatment. Among which, 16 cases of patients (19 feet) received open plantar fascia release, and the other 15 cases of patients (20 feet) received percutaneous radiofrequency ablation. There were 12 male and 19 female patients with a male-to-female ratio of 1:1.58. The average age of the enrolled 31 patients was 52.13 years old. Twenty-six of the patients were at the age of 40–70 years old. There were no differences of patient’s demographic characteristics between the two groups including ages, sex, body mass index (BMI), and duration of pain (Table 1). The average duration of the symptoms was 20.03 months (ranging from 6 to 120 months) in all patients. In addition, there were 18 cases of patients presented with calcaneal osteophytes. The average operative time was 36.78 min and 19.73 min in the open plantar fascia release and the percutaneous radiofrequency ablation, respectively. The radiofrequency ablation group had a shorter mean operative time than the open group (*P* = 0.012). The average time that patients recovered to normal activity was 25.94 days in the plantar fascia open release group and 13.27 days in the percutaneous radiofrequency ablation group. The recovery time of the percutaneous radiofrequency ablation treated group was shorter than the open release group (*P* = 0.008) (Table 2).

**Table 1:** Patient’s demographics of the two groups

Variable	Open plantar fascia release	Percutaneous radiofrequency ablation	*P* value
Age, y			
Mean ± SD	49.63 ± 9.78	54.80 ± 13.83	0.236
Range	25–65	24–77	
Sex, no.			
Male	6	6	0.891
Female	10	9	0.891
BMI, kg/m^2^			
Mean ± SD	26.80 ± 2.67	26.27 ± 3.03	0.604
Range	21.48–32.11	21.72 ± 32	
Duration of pain, mo			
Mean ± SD	22.75 ± 30.97	17.13 ± 12.70	0.878
Range	6–120	6–60	

*y* years, *BMI* body mass index, *no*. numbers, *mo* months

**Table 2:** Operative time and recovery time of the two groups

	Operative time (min)	Recovery time (days)
Open plantar fascia release	36.78 ± 22.62	25.94 ± 15.47
Percutaneous radiofrequency ablation	19.93 ± 9.66	13.27 ± 7.48
*P* value	0.012	0.008

According to the results of last follow-up, the patients in the open plantar fascia release group reported a preoperative and postoperative mean VAS scores of 8.81 ± 1.11 and 0.50 ± 1.41, respectively, with an average reduction of 8.31 points (*P* < 0.001). The patients in the percutaneous radiofrequency ablation group had mean VAS scores of 7.87 ± 1.73 and 0.73 ± 1.28 preoperatively and postoperatively. The average reduction was 7.14 points (*P* < 0.001) (Table 3).

**Table 3:** VAS scores of the two groups

	Preoperative VAS score	Postoperative VAS score	*P* value
Open plantar fascia release	8.81 ± 1.11	0.50 ± 1.41	0.000
Percutaneous radiofrequency ablation	7.87 ± 1.73	0.73 ± 1.28	0.000
*P* value	0.078	0.634	

AOFAS scores were also obtained in patients of the open release group with a mean AOFAS score of 39.63 ± 8.52 and 99.38 ± 2.50 before and after operation. An average of 59.75 points (*P* < 0.001) increase was found. The mean AOFAS scores in the patients treated with percutaneous radiofrequency ablation were 42.73 ± 10.75 and 98.40 ± 4.24 before and after operation with an increase of average 56.67 points (*P* < 0.001) (Table 4). There is no significant difference between the two groups in AOFAS points postoperatively in the follow-up.

**Table 4:** AOFAS scores of the two groups

	Preoperative AOFAS score	Postoperative AOFAS score	*P* value
Open plantar fascia release	39.63 ± 8.52	99.38 ± 2.50	0.000
Percutaneous radiofrequency ablation	42.73 ± 10.75	98.40 ± 4.24	0.000
*P* value	0.378	0.438	

All 31 patients were followed up by telephone questionnaire, and 29 patients were satisfied with the operation. Two patients with poor satisfaction were all in the open plantar fascia release group. In these two cases, 1 patient had a combined insertional Achilles tendinopathy. However, the plantar fascia pain was completely relieved after operation except for the pain of the Achilles tendinopathy. The other case had a flatfoot deformity before the procedure and sustained irritation of subtalar screw from other procedures of flatfoot. No complications of infection, hematoma, and complex regional pain syndrome occurred in all 31 patients.

## Discussion

Heel pain is a general term of various disease processes affecting the daily work and life of patients. The etiologies are complicated and include plantar fasciitis, rupture of plantar fascia, calcaneal stress fracture, heel fat pad atrophy and/or inflammation caused by strain and degeneration, retrocalcaneal bursitis, Achilles tendinitis, and irritation of calcaneal osteophytes formation. Many support that plantar fasciitis is one of the main causes. Epidemiological studies show that plantar fasciitis is estimated to account for 11–15% of all foot diseases that require medical attention [16]. It is common in the elderly population aged from 40 to 70 years. Workers with long standing hours, runners, and obese patients with BMI more than 30 kg/m^2^ are the high-risk population with higher intensity of pain [17].

It is estimated that about 90% of patients with plantar fasciitis received conservative treatment with satisfactory effect, and no further surgical intervention was needed [18, 19]. Therefore, the American Orthopaedic Foot and Ankle Society recommends that patients diagnosed with plantar fasciitis should receive at least 6 months of conservative treatment before undergoing surgical intervention [8]. Conservative treatment methods include stretching traction training, splinting, bracing, extracorporeal shock wave, oral administration of drugs, and local injections of corticosteroid medications. Drug injection is also a common treatment for fasciitis. Rastegar et al. found that steroid injection could palliate plantar heel pain rapidly but dry needling can provide more satisfactory results for patients with plantar fasciitis in the long term in a random clinical trial [20]. PRP injection was associated with improved pain and function scores at 3-month follow-up when compared with corticosteroid injections [21, 22]. Polydeoxyribonucleotide was certificated as an effective and safe treatment option and may be considered for plantar fasciitis by Kim and Chung [23].

For patients with intractable plantar fasciitis failed at least 6 months of conservative treatment, surgical intervention can be considered. Open plantar fascia release is the most traditional operation method. According to the reports, the postoperative satisfaction rate of open release was 50–95% [24, 25]. However, disadvantages of the surgery include large wound, longer postoperative recovery time, and potential postoperative occurrence of complex regional pain syndrome. Although plantar fascia release under arthroscopy has the advantage of being minimally invasive, there are still postoperative complications mostly reported with incomplete pain relieving occurring higher in rate than the traditional open plantar fascia release [25–28]. Xu et al. reported a modified minimally invasive surgical system in the open release of plantar fascia [29]. In this study, all of the patients in the open plantar fascia release were operated with this modified minimally invasive technique.

Bipolar radiofrequency ablation technique was first used in the treatment of cardiovascular disease to promote the regeneration of ischemic myocardium in patients with chronic heart failure [30–32]. Weil and his colleagues applied this technique for the treatment of intractable plantar fasciitis in the early stage with good results [33]. In a prospective study consisting of 21 cases in 2011, Sorensen et al. supported the curative effect of this type of technique. In the same year, Hormozi et al. also reported the therapeutic effect of this operation based on a prospective study of 14 cases [34, 35].

In this study, the average age of the enrolled 31 patients was 52.13 years old, among which, there were 26 patients at the age of 40–70 years old accounting for 83.87% of the total subjects which was similar to the demographics reported from the previous investigations. There were 14 overweight patients (24 ≤ BMI < 28) and 10 obese patients (BMI ≥ 28), accounting for 45.16% and 32.26%, respectively.

The results of this study showed a shorter average operation time of percutaneous radiofrequency ablation than the open plantar fascia release. We contribute this result to the relatively simple operation process of this method as previously reported [34, 35]. At the same time, the average recovery time was shorter in the percutaneous radiofrequency ablation group, and the result was also consistent with the previous literature [34]. Possible reasons might be that percutaneous radiofrequency ablation had relatively smaller individual wounds and thus retained the integrity of the plantar fascia [35]. There was no difference of postoperative VAS scores and the AOFAS-AH scores between the two groups, indicating that the two types of surgical procedures share the same curative effects. There were no major postoperative complications in both groups in our study.

Calcaneal osteophytes are also known as calcaneal spur. Many scholars believed that the calcaneal osteophytes are one of the major factors responsible for heel pain, and some scholars even described heel pain as calcaneal osteophyte syndrome [1]. Johal and Milner in their study insisted that there was a positive correlation between calcaneal osteophytes and plantar fasciitis. However, the hypothesis was not able to explain the asymptomatic calcaneal osteophytes [36]. Kumai and Benjamin supported that calcaneal osteophytes were associated with the degeneration of cartilage cells at the insertion of the plantar fascia, regardless of the role of the traction of the plantar fascia [37]. In this study, 18 cases out of 31 had calcaneal osteophytes, of which only 1 case underwent intraoperative calcaneal osteophyte resection. All patients had significant improvement with pain postoperatively. We may have speculated that there was no significant correlation between calcaneal osteophytes and the incidence of heel pain.

During the follow-up period, 29 patients were satisfied with the operation with a satisfaction rate of 93.55%. Two patients reported poor clinical results. Among which, 1 patient had a combined Achilles tendinopathy. The pain from plantar fascia was completely relieved after operation while there was still pain of the Achilles tendinopathy. The other patient had a flatfoot deformity treated before the plantar fascia operation. The irritation of the subtalar tarsal screw remained after our intervention of the plantar fascia. However, heel pain symptoms and limb function were both improved in the two patients after plantar fascia operation.

With the design limitation of a retrospective study, our results could be affected by various factors, such as selection bias, small sample size, and lack of long-term follow-up period, compared with randomized clinical trials [38]. On the other hand, small sample size is common in many of the available reports. The shield could protect surrounding tissues when plantar fascia were cut compared with the way published by Oliva et al. [39]. Percutaneous radiofrequency ablation is a new technique of intractable plantar aponeurositis. We believe that the results of this study will be helpful for relevant design of prospective studies in the future. Compared with conservative treatments such as extracorporeal shock wave therapy [40], these two surgeries are kinds of invasive ways to cure intractable plantar aponeurositis which means these techniques will only be used when conservative treatments show limited effects.

Collectively, based on the experiences in the treatment of 31 patients with intractable plantar fasciitis, we believe that open plantar fascia release and percutaneous radiofrequency ablation can significantly relieve the pain and improve limb function in patients with intractable plantar fasciitis. Percutaneous radiofrequency ablation has shown the advantages of shorter operation time, shorter postoperative recovery time, and higher postoperative function scores and therefore may be a better option while selecting surgical interventions of the intractable plantar fasciitis.

## Conclusion

The observations made in the present study suggest that the treatment of plantar fasciitis with plantar fascia lysis or percutaneous radiofrequency ablation is equally effective. The technique of percutaneous radiofrequency ablation can shorten the operative time and postoperative recovery time.

## Data Availability

All datasets used during the current study are available from the corresponding author on reasonable request.

## Abbreviations

AOFAS-AH: American Orthopaedic Foot and Ankle Society Ankle-Hindfoot Scale
BMI: Body mass index
VAS: Visual analog scale
